# The *Xanthomonas* RaxH-RaxR Two-Component Regulatory System Is Orthologous to the Zinc-Responsive *Pseudomonas* ColS-ColR System

**DOI:** 10.3390/microorganisms9071458

**Published:** 2021-07-07

**Authors:** Valley Stewart, Pamela C. Ronald

**Affiliations:** 1 Department of Microbiology & Molecular Genetics, University of California, Davis, CA 95616, USA; 2 Department of Plant Pathology and Genome Center, University of California, Davis, CA 95616, USA; pcronald@ucdavis.edu

**Keywords:** ColS-ColR, RaxH-RaxR, VgrS-VgrR, lipid A remodeling, lipid A phosphoethanolamine transferase, lipid A 1-phosphatase, *Xanthomonas oryzae* pv. *oryzae*

## Abstract

Genome sequence comparisons to infer likely gene functions require accurate ortholog assignments. In *Pseudomonas* spp., the sensor-regulator ColS-ColR two-component regulatory system responds to zinc and other metals to control certain membrane-related functions, including lipid A remodeling. In *Xanthomonas* spp., three different two-component regulatory systems, RaxH-RaxR, VgrS-VgrR, and DetS-DetR, have been denoted as ColS-ColR in several different genome annotations and publications. To clarify these assignments, we compared the sensor periplasmic domain sequences and found that those from *Pseudomonas* ColS and *Xanthomonas* RaxH share a similar size as well as the location of a Glu-X-X-Glu metal ion-binding motif. Furthermore, we determined that three genes adjacent to *raxRH* are predicted to encode enzymes that remodel the lipid A component of lipopolysaccharide. The modifications catalyzed by lipid A phosphoethanolamine transferase (EptA) and lipid A 1-phosphatase (LpxE) previously were detected in lipid A from multiple *Xanthomonas* spp. The third gene encodes a predicted lipid A glycosyl transferase (ArnT). Together, these results indicate that the *Xanthomonas* RaxH-RaxR system is orthologous to the *Pseudomonas* ColS-ColR system that regulates lipid A remodeling. To avoid future confusion, we recommend that the terms ColS and ColR no longer be applied to *Xanthomonas* spp., and that the Vgr, Rax, and Det designations be used instead.

## 1. Introduction

Diverse *Xanthomonas* species and pathovars cause serious diseases in most plants [1,2]. One example is *X. oryzae* pv. *oryzae* (*Xoo*), which causes significant rice crop losses in Asia and Africa [3]. Resistance to *Xoo* is conferred by the rice XA21 receptor kinase [4], which activates innate immune responses upon binding the *Xoo* RaxX (required for activation of XA21 component X) protein [5]. RaxX is a secreted sulfopeptide similar to the plant PSY sulfopeptide growth hormone [6,7], and is synthesized by some but not all *Xanthomonas* spp. [8].

**Figure 1:**
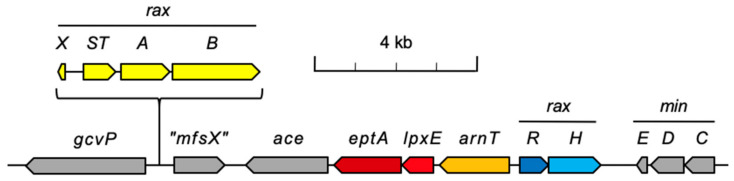
Genes in the *Xoo* strain PXO99^A^ *gcvP-minCDE* region. Drawn to scale. Gene symbols are *eptA*, phosphoethanolamine transferase (dark red); *lpxE*, lipid A 1-phosphatase (red); *arnT*, sugar-undecaprenyl phosphate transferase (orange); *raxR* (dark blue); *raxH* (light blue). Other gene symbols are described in the text. The *raxX-raxSTAB* gene cluster is between *gcvP* and “*mfxS*” only in certain *Xanthomonas* species (Table S1). Small open reading frames with unknown function are not shown. See text for references.

*Xoo* proteins required specifically for RaxX processing and secretion are encoded by the *raxX-raxSTAB* gene cluster [5,9,10,11] (Figure 1). These genes are adjacent to a seven-gene cluster including the *raxHR* genes [12], which encode the sensor histidine kinase and response regulator proteins, respectively, of a two-component regulatory system [12,13]. Phosphoryl transfer from a sensor to its cognate response regulator increases DNA binding in order to control target gene transcription initiation [14,15,16]. Truncated soluble RaxH protein rapidly autophosphorylates and catalyzes efficient phosphoryl-transfer to RaxR protein in vitro [13] as expected for a cognate two-component regulatory pair [17]. To date, the RaxR protein has not been demonstrated to bind DNA or activate transcription.

*Pseudomonas* spp. encode the ColS-ColR two-component regulatory system, identified initially in *P. fluorescens* as required for root colonization through controlling functions that remodel LPS (lipopolysaccharide) [18,19]. In *Xanthomonas* spp., three separate two-component regulatory pairs have been denoted as ColS-ColR in several different published genome sequences [20,21,22,23,24,25,26] and research papers [27,28,29,30]. Separately, each of these *Xanthomonas* ColS-ColR-like pairs has been assigned unique symbols based on experimental analysis: RaxH-RaxR [12], VgrS-VgrR (virulence and growth regulator) [31], and DetS-DetR (detoxifying) [32].

In *Xoo*, the RaxH-RaxR pair may play a role in setting the level of RaxX synthesis, processing, or secretion through a mechanism not yet determined [12]. In *Xoo*, *Xanthomonas campestris* pv. *campestris* (*Xcc*), and *X. citri* (*Xac*), the VgrS-VgrR pair controls virulence, the hypersensitive response, environmental stress tolerance, and growth with limited iron [27,28,29,31]. The DetR response regulator is required for virulence in *Xoo* [32] but not in *Xcc* [27]. Unlike *Pseudomonas* ColS-ColR, neither VgrS-VgrR nor DetS-DetR has been implicated in the control of lipid A remodeling enzyme synthesis.

Orthologs are genes derived from the most recent common ancestor of the lineages being considered [33], and orthologous gene products generally perform similar functions [34,35]. To advance understanding of the *raxRH* and adjacent genes (Figure 1), we determined their orthologous relationships with genes of known function from other genera. These determinations generated two conclusions: first, that the *Xanthomonas* RaxH-RaxR pair is orthologous to the *Pseudomonas* ColS-ColR pair, which regulates lipid A remodeling enzyme synthesis in response to excess Zn^2+^ [36,37]; second, that the *Xanthomonas* genes denoted here as *eptA*, *lpxE*, and *arnT* likely encode enzymes that remodel lipid A in response to cell envelope stress. These results suggest that the *Xanthomonas* RaxH-RaxR two-component system regulates expression of the *arnT-lpxE-eptA* gene cluster encoding lipid A remodeling enzymes.

## 2. Materials and Methods

### 2.1. Sequence Analyses

The BLAST programs [38,39] were used for database searches and for determining reciprocal best hits. Nucleotide and deduced amino acid sequences were edited and analyzed with the programs EditSeq™ and MegAlign™ (version 14.1.0), DNASTAR, Madison, WI, USA. The Integrated Microbial Genomes interface [40] was used to compare genome segments from different species in order to analyze genome neighborhoods.

*Xanthomonas* genes described here in detail are from *Xoo* strain PXO99^A^ (GenBank accession NC_010717.2) except as noted otherwise. Genes, locus tags and accession numbers are presented in Table S1.

### 2.2. Ortholog Identification

Orthologous genes were identified initially by analyzing their predicted amino acid sequences for reciprocal BLASTP best hits [34,35] using default parameters for database BLASTP searches [41] (expect = 10; word size = 6; matrix = BLOSUM62; gap costs = existence 11, extension 1; no compositional adjustments). Initial searches focused on comparisons between genes from *Xoo* and from *Pseudomonas aeruginosa*, a well-annotated close relative [42]. Other species and genera were examined as needed. Candidate orthologous pairs were then evaluated to ensure that they have similar lengths, that sequence similarity extends throughout most of their lengths, and that they have similar domain compositions. Candidates were further examined for the presence of conserved and functionally-important residues and for genome neighborhood.

Pairwise comparisons generated for Table 1 were performed with the pairwise BLASTP function using default parameters (expect = 0.05; word size = 3; matrix = BLOSUM62; gap costs = existence 11, extension 1; conditional compositional score matrix adjustment).

### 2.3. Predicted Transmembrane Helices

Sequences were evaluated in the TMHMM Server v. 2.0 accessed through the Danish Technical University (http://www.cbs.dtu.dk/services/TMHMM/, accessed on 14 April 2021). Predictions were refined by manual adjustment as necessary.

## 3. Results

### 3.1. The gcvP-minCDE Region in the Xoo Strain PXO99^A^ Genome

The *raxX-raxSTAB* gene cluster apparently has been gained and lost multiple times during *Xanthomonas* speciation [8]. In all cases identified [8], this cluster is located in the same genome neighborhood, between the *gcvP* gene encoding a subunit of glycine dehydrogenase [43] on one side, and the *minCDE* operon encoding proteins required for cell division [44] on the other (Figure 1). The gene denoted as *“mfsX”* is predicted to encode a major facilitator subfamily permease [8]. The *eptA*, *lpxE*, and *arnT* genes are predicted to encode enzymes involved in lipid A remodeling, as described below (Section 3.3, Section 3.4 and Section 3.5).

The *ace* gene, identified first in *Xac*, encodes a dipeptidyl-carboxypeptidase homologous to mammalian ACE (angiotensin converting enzyme). *Xac* ACE enzyme efficiently digests mammalian ACE substrates such as angiotensin [45], although the authentic substrate(s) and physiological function(s) for bacterial ACE are not known. *ace* gene homologs are distributed throughout diverse bacterial phyla [45], and gene neighborhoods are not conserved across different genera (not shown).

The *raxR-raxH* and *lpxE-eptA* gene pairs have overlapping termination and initiation codons (ATGTCTAA and ATGA, respectively). Such overlaps indicate that the two gene products function together [46]. Otherwise, the *raxR-arnT* intergenic region is 218 nt, the *arnT-lpxE* intergenic region is 99 nt, and the *eptA-ace* intergenic region is 111 nt (Figure 1).

### 3.2. Evidence That Xanthomonas RaxH-RaxR and Pseudomonas ColS-ColR Are Orthologous Pairs

The two-component regulatory systems considered here all resemble the model EnvZ-OmpR pair that controls outer membrane porin synthesis in *Escherichia coli* [17,47]. In EnvZ-OmpR-type systems, the sensor protein comprises an amino-terminal periplasmic signal input domain and a carboxyl-terminal cytoplasmic transmitter module with histidine autokinase activity. The response regulator protein has amino-terminal phospho-accepting receiver and carboxyl-terminal DNA-binding domains. About 11 distinct OmpR-type response regulators [48] are conserved across different *Xanthomonas* spp. [49,50]. Of these, three cognate sensors have EnvZ-type transmitter sequences (HPK2 family [51]): RaxH, VgrS, and DetS. All three sensors also have been denoted as ColS (see Introduction), based on sequence similarities across the transmitter domains.

**Figure 2:**
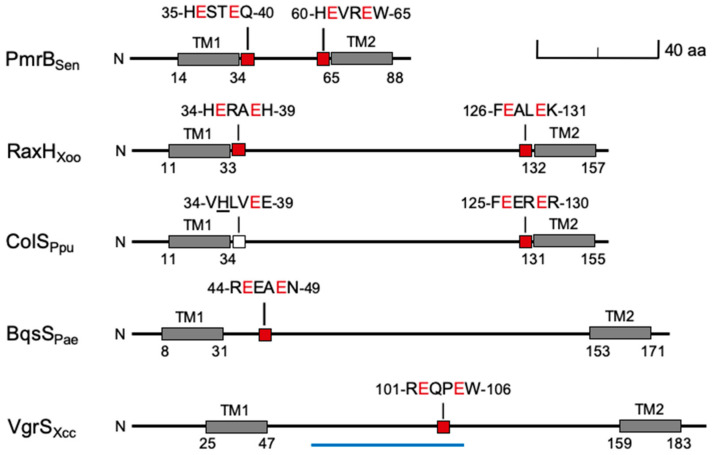
Periplasmic domain schematic structures for selected sensor proteins. Drawn to scale. The amino-terminal sequences are illustrated through the second of two transmembrane (TM) helices, shown as gray filled boxes. Numbers show positions of specific residues. The conserved ExxE motifs are shown as red-filled boxes, with the corresponding sequences above. The variant HxxE motif in ColS is shown as a white box. The blue line under the *Xcc* VgrS periplasmic domain shows the extent of a conserved sequence substitution in the *Xoo* sequence that removes the ExxE motif. Sen, *Salmonella enterica* LT2, Xoo, *Xanthomonas oryzae* pv. *oryzae* PXO99^A^, Ppu, *Pseudomonas pudita* KT2440, Pae, *Pseudomonas aeruginosa* PAO1, Xcc, *Xanthomonas campestris* pv. *campestris* 8004. See text for references.

Three criteria indicate that the *Xanthomonas* RaxH-RaxR pair is orthologous to the *Pseudomonas* ColS-ColR pair [34,35]. First, the RaxH and *Pseudomonas* ColS periplasmic domain sequences display virtually identical size and predicted topology, even though they share only 20% identical residues (Figure 2 and Figure S1). The other two candidate ColS orthologs (VgrS and DetS) are predicted to adopt similar topologies, but their periplasmic domain sequences are different lengths (Figure 2 and not shown).

It should be noted that relatively low sequence identity between orthologous sensor periplasmic domains is typical. For example, the PhoQ sensors from *P. aeruginosa* [52] and *Xoo* [53] share only 25% sequence identity between the periplasmic domains, in contrast to 42% between the transmitter domains and 56% between the corresponding PhoP response regulators (alignments not shown).

Second, the ColS sequence includes the critical metal ion-binding ExxE (Glu-X-X-Glu) motif, just proximal to the second transmembrane helix, which is required for responses to Fe^3+^, Zn^2+^ and other metal ions [36]. This motif is at the same position in RaxH (Figure 2 and Figure S1), whereas the VgrS Fe^3+^-binding ExxE motif [54] is in the middle of the periplasmic domain sequence (Figure 2). (The DetS periplasmic domain sequence does not contain an ExxE motif.) For comparison, Figure 2 depicts periplasmic domains from two other ExxE motif-containing sensors, *Salmonella enterica* PmrB, which signals in response to excess Fe^3+^ or Al^3+^ [55], and *P. aeruginosa* BqsS, which signals in response to excess Fe^2+^ [56].

Remarkably, the RaxH sequence also includes a second ExxE motif, just distal to the first transmembrane helix, that is not conserved in ColS sequences (Figure 2 and Figure S1). In this respect, the RaxH sequence is similar to that of PmrB [55] (Figure 2). These differences in ExxE motif numbers and locations imply differences in binding specificity and/or affinities for different metal ions.

The third criterion for assigning RaxH-RaxR and ColS-ColR as orthologous pairs comes from analyzing genome neighborhoods [35]. Both the *Xanthomonas raxRH* and *Pseudomonas colRS* genes are adjacent to the divergently transcribed *lpxE* ortholog (Section 3.6, below). These neighborhoods differ in that the *Pseudomonas eptA* and *arnT* genes are elsewhere in the genome. Nonetheless, in *P. aeruginosa* and *P. putida*, the ColS-ColR pair activates expression of the *eptA* [37] and predicted *lpxE* genes [36,57], and represses expression of the *arnT*-containing *arnB* operon [37], all in response to excess Zn^2+^. Accordingly, the *Xanthomonas eptA-lpxE-arnT* cluster immediately adjacent to the *raxRH* genes suggests independently that RaxH-RaxR and ColS-ColR control similar physiological functions [35].

### 3.3. The Xanthomonas eptA Gene Is Predicted to Encode Lipid A Phosphoethanolamine Transferase

Section 3.3, Section 3.4 and Section 3.5 concern the genes *eptA*, *lpxE*, and *arnT*, predicted to encode lipid A remodeling enzymes and to be regulated by the RaxH-RaxR two-component system.

**Figure 3:**
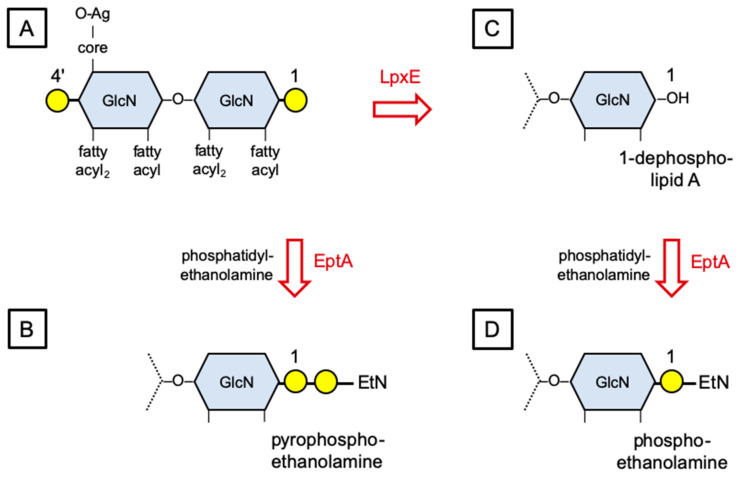
EptA (phosphoethanolamine transferase) and LpxE (lipid A 1-phosphatase) activities. (**A**) Lipid A schematic structure. The glucosamine disaccharide is decorated with six fatty acyl chains, the core oligosaccharide that ligates the polymorphic O-antigen (O-Ag) polysaccharide, and phosphate esters at positions 1 and 4’. (**B**) EptA enzyme adds phosphoethanolamine (P-EtN) to form a pyrophosphoethanolamine moiety. Only one glucosamine from the lipid A disaccharide is shown, but the same reaction also can occur at the 4’ position. (**C**) LpxE enzyme removes phosphate-1. (**D**) EptA enzyme adds P-EtN to form a phosphoethanolamine moiety. See text for references.

LPS consists of the membrane anchor lipid A connected through a core oligosaccharide to the polymorphic O antigen polysaccharide [58,59,60]. Lipid A in many gram-negative genera (including Xanthomonas) is an acylated β-1′,6-linked glucosamine disaccharide bis-phosphate [61,62,63,64] (Figure 3A). The phosphates at positions 1 and 4′ are bridged by Mg^2+^ ions, providing one of the LPS lateral interactions necessary to form the characteristic tight permeability barrier [60,65].

As the outer membrane’s external surface, LPS confers unique permeability properties while also providing targets for bacteriophage attachment, host pattern recognition, and antimicrobial peptides. Accordingly, LPS structures can be remodeled not only by changing the O antigen but also through modifications to the core oligosaccharide and lipid A components [58,59,60]. Lipid A remodeling enzyme synthesis is induced by certain disruptive envelope stresses, including cation imbalance and cationic antimicrobial peptides.

One widespread modification is catalyzed by lipid A phosphoethanolamine transferase (EptA enzyme), which acts in the periplasm to transfer phosphoethanolamine (P-EtN) from phosphatidylethanolamine to positions 1 and/or 4′ on the lipid A disaccharide [58] (Figure 3B). The *Xoo* putative EptA sequence shares ca. 40% identity over most of its length with the orthologous *P. aeruginosa* EptA sequence (Table 1). Sequence alignments (not shown) reveal that the Xoo putative EptA sequence includes critical residues identified in *N. meningitidis* EptA [66]: Glu-240, Thr-280, Asp-452 and His-453 (Glu-253, Thr-293, Asp-463 and His-464 in *Xoo* EptA), which form the Zn^2+^-binding active site, and disulfides formed by Cys residue pairs at positions 276–286, 327–331, 348–353, 402–410, and 499–540 (287–297, 338–342, 359–364, 413–421, and 510–549 in *Xoo* EptA).

Lipid A from wild-type *Xanthomonas campestris* pv. *campestris* (*Xcc*) has undetectable EtN [63], whereas lipid A from a nonpathogenic *rfaX* mutant displays nonstoichiometric substitution with EtN at positions 1 and/or 4′ on the lipid A disaccharide [67]. The *rfaX* lesion generates LPS with a severely truncated core oligosaccharide, and so the resulting outer membrane disruption likely induces EptA synthesis [67]. Lipid A with linked EtN also has been detected in *X. campestris* pv. *pruni* [68], *Xac* [61], and *Xoo* [62], although not in *X. fragariae* [68] or *X. translucens* pv. *translucens* [64]. These results provide evidence that the *Xoo* putative EptA enzyme is synthesized and active (Figure 3).

### 3.4. The Xanthomonas lpxE Gene Is Predicted to Encode Lipid A 1-phosphatase

A second lipid A remodeling reaction is catalyzed by lipid A 1-phosphatase (LpxE enzyme), which functions in the periplasm [69,70] (Figure 3C). The *Xoo* putative LpxE sequence shares ca. 40% identity with the orthologous *P. aeruginosa* putative *LpxE* sequence (Table 1). These sequences share similar predicted membrane topology with the *Rhizobium etli* LpxE sequence [69,70], each having six predicted transmembrane helices delimiting three periplasmic segments and four cytoplasmic segments (alignments not shown). Neither the structure nor the active site for LpxE enzyme has been determined. However, the *Xoo* putative LptE sequence includes several residues conserved in *R. etli* LpxE thought to be important for catalysis [69]: Lys-123, Pro-131, Pro-155, Gly-157, His-158 and His-197 (Lys-127, Pro-135, Pro-164, Gly-166, His-167, and His-213 in *Xoo* LpxE).

Some LpxE homologs have phosphatase activity on substrates other than lipid A [69,70]. Three lines of evidence support the hypothesis that the *Xoo* putative LpxE enzyme is lipid A 1-phosphatase. First, *Helicobactri pylori* EptA and LpxE enzymes are expressed from the *eptA-lpxE* operon [71], revealing a shared genome neighborhood. Recall that the *Xoo lpxE* and *eptA* genes overlap, indicating likely translational coupling (Figure 1; Section 3.1, above).

Second, this genetic association reflects functional association, as *H. pylori* LpxE removes the lipid A 1-phosphate before EptA adds P-EtN, thereby forming a monophosphoethanolamine (P-EtN) moiety [72] (Figure 3C,D). In many other studied genera, P-EtN is added to the existing phosphate group, thereby forming a pyrophosphoethanolamine (PP-EtN) moiety [58,60] (Figure 3B). Strikingly, lipid A from *Xac* is a mixture wherein some molecules contain two PP-EtN moieties and others contain one PP-EtN and one P-EtN [61], providing evidence that *Xac* lipid A has been dephosphorylated in some cases before P-EtN addition.

Third, *H. pylori* and *R. etli* both synthesize dephospho-lipid A through LpxE (and LpxF) activities [73,74]. Some lipid A molecules from *X. campestris* pv. *pruni* are dephosphorylated [68], providing evidence that the *Xanthomonas* putative LpxE enzyme is synthesized and active (Figure 3). In *H. pylori* and *R. etli*, LpxE-dependent lipid A 1-dephosphorylation confers some resistance to antimicrobial peptides [71,75], although higher-level resistance requires LpxF-dependent lipid A 4′-dephosphorylation as well [73,74].

### 3.5. The Xanthomonas arnT Gene Is Predicted to Encode Lipid A Glycosyl Transferase

A third lipid A remodeling reaction is catalyzed by lipid A glycosyl transferase (ArnT enzyme), which functions in the periplasm to transfer 4-amino-4-deoxy-L-arabinose (Ara4N) from its undecaprenyl phosphate carrier to the phosphate at either end of the lipid A disaccharide [58,59,60]. The enzyme comprises an amino-terminal domain with 13 transmembrane helices and a carboxyl-terminal periplasmic domain whose sequence is poorly-conserved [76,77]. The *Xoo* putative ArnT sequence shares ca. 30% identity with the orthologous *P. aeruginosa* ArnT sequence over the amino-terminal half (Table 1), similar to other ArnT sequence comparisons [76,77]. Sequence alignments (not shown) reveal that the *Xoo* putative ArnT sequence includes critical residues identified in *Cupriavidus metallidurans* ArnT [76]: Glu-84, Lys-85, Arg-270 and Tyr-345 (Glu-70, Lys-71, Arg-264 and Tyr-335 in *Xoo* ArnT), all involved in coordinating the phosphate group of the undecaprenyl carrier, and the ion pairs Asp-55/Arg-58 and Asp-158/Lys-203 (Asp-41/Arg-44 and Asp-144/Lys-185 in *Xoo* ArnT), which orient the lipid A phosphate for catalysis.

ArnT enzyme first was identified and characterized in enterobacteria, where it is expressed from the *arnBCADTEF* operon [55]. Besides *arnT*, the remaining six *arn* genes encode enzymes that synthesize and translocate the Ara4N-undecaprenyl phosphate sugar donor for ArnT catalysis [78]. However, in some other species, the ArnT enzyme instead transfers galactosamine, glucosamine, or glucose [79,80,81]. For example, *Francisella novicida* encodes two homologs of the enterobacterial *arnC* gene, encoding undecaprenyl phosphate sugar transferase that instead are specific for galactosamine (FlmF2) or glucose (FlmF1) [82].

The enzyme responsible for making the putative undecaprenyl phosphate sugar substrate for *Xanthomonas* ArnT enzyme is not clear. At least two genes, both unlinked to the *gcvP-minCDE* cluster, potentially encode ArnC-like enzymes. Gene PXO_RS22265 encodes a protein ca. 30% identical to *P. aeruginosa* ArnC, although it is only about two-thirds as long (Table 1). This gene is in a cluster between PXO_RS22260, which encodes a predicted sugar-nucleotide epimerase, and PXO_RS22270, which encodes a ca. 100 residue protein homologous to a biochemically-uncharacterized amino-terminal domain in the LpxB lipid A-disaccharide synthase from *Chlamydia* and related genera (accession WP_009873791.1). Indeed, PXO_RS22265 and PXO_RS22270 homologs are linked to *arnT* genes in other species (Section 3.6 below), suggesting that they encode functions involved in lipid A remodeling. However, these genes also are present in species, such as clade 1 *Xanthomonas* spp., for which we did not identify an *arnT* ortholog (Section 3.6 below). Separately, gene PXO_RS14680 encodes a protein ca. 37% identical to the *F. novicida* FlmF1 and FlmF2 sequences (Table 1). However, the adjacent gene PXO_RS14685 encodes an enzyme likely involved in peptidoglycan biosynthesis, so the PXO_RS14680-encoded protein may function in this process instead.

Thus, it is plausible that the *Xanthomonas* ArnT enzyme catalyzes transfer of a sugar other than Ara4N to lipid A. Nevertheless, to our knowledge, the only reported modifications to *Xanthomonas* lipid A disaccharide phosphates are the dephosphorylation and the P-EtN and PP-EtN moieties described above. Accordingly, predicted function(s) for the *Xoo* putative ArnT enzyme are unspecified. Perhaps lipid A analysis from cultures grown under different conditions will identify a plausible ArnT-dependent modification.

### 3.6. raxRH Gene Neighborhoods in Members of the Order Xanthomonadales (Lysobacterales)

Expanding on our prior analysis [8], we compared the *gcvP-minCDE* regions across the range of *Xanthomonas* spp. There were 31 correctly-named *Xanthomonas* spp. as of 20 May 2021 [83]. We used the Integrated Microbial Genomes platform [40] to examine 41 genomes, with each correctly-named *Xanthomonas* species included at least once, to determine their *raxRH* gene neighborhoods (Table S2). The *gcvP-minCDE* region is virtually identical in each case (not shown), with two exceptions. First, some strains have the *raxX-raxSTAB* gene cluster between genes *gcvP* and “*mfsX*” (Figure 1), whereas others do not [8] (Table S2). Second, the relatively small number of strains from clade 1 [1,2] (Table S2) specifically lack only the *arnT* gene (Figure S2).

This phylogenetic distribution indicates that the complete *gcvP-minCDE* region (excepting the *raxX-raxSTAB* gene cluster [8]) was not acquired recently through lateral gene transfer [84]. A separate, relatively simple criterion to examine this point is provided by the G+C percentage, which often differs in laterally transferred genes [84]. In *Xoo* PXO99^A^, the G+C percentage is relatively constant across the *gcvP-minCDE* region (including the *raxX-raxSTAB* genes) and close to the median value for the entire genome: 63.7% (Figure S3).

Next, we used BLAST to search for homologs of the *Xoo* PXO99^A^ RaxH periplasmic domain sequence in other species from the order *Xanthomonadales* (renamed as *Lysobacterales* [83]). This search identified sequences from a variety of genera in the family *Xanthomonadaceae* (renamed as *Lysobacteraceae* [83]) and two genera from the family *Rhodanobacteraceae* (Table S1). The alignment of representative RaxH periplasmic domain sequences (Figure S1) shows that all share an overall length, predicted membrane topology, and conserved sequence features with *Xoo* RaxH. Notably, however, the TM2-proximal ExxE motif is not fully conserved, with the first Glu replaced by Gln, His, or Thr in some sequences (Figure S1 and not shown).

Finally, we examined *raxRH* gene neighborhoods for these and related strains. In each case, the *raxRH* genes are adjacent to one or another combination of the *arnT*, *lpxE*, and *eptA* genes, reinforcing the association of the RaxH-RaxR two-component regulatory system with functions for LPS remodeling (Figure S2).

## 4. Discussion

### 4.1. RaxH-RaxR and ColS-ColR Are Orthologous Two-Component Regulatory System Pairs

Sequence comparisons between different phylogenetic groups can provide indispensable guidance for predicting gene functions. However, useful predictions depend upon accurate ortholog assignments [34,35]. Otherwise, genes with the same designations might be considered to have the same functions in different genera, potentially obscuring meaningful differences.

The ColS-ColR two-component regulatory system in *Pseudomonas* spp. responds to metal ions such as Zn^2+^ to control genes whose products remodel lipid A [18,19,36,37,57]. In *Xanthomonas* spp., the designation ColS-ColR has often [27,28,29,30] but not always [49,54,85] been applied to the VgrS-VgrR two-component regulatory system that controls a variety of virulence-related functions. However, evidence presented here suggests that the *Xanthomonas* RaxH and RaxR proteins are the true orthologs of ColS and ColR. Therefore, conflating *Xanthomonas* VgrS-VgrR with *Pseudomonas* ColS-ColR is potentially misleading, especially since the two regulatory systems appear to control different functions.

Although the RaxH and ColS sequences share overall similarities, they may have potential functional differences, for example in the number of ExxE motifs (Figure S1). Therefore, we recommend that the ColS and ColR designations no longer be applied to *Xanthomonas* two-component regulatory systems, and that the appropriate Rax, Vgr, and Det designations be used instead.

### 4.2. Two-Component Regulatory Systems Regulate Lipid A Remodeling

In other species, lipid A remodeling enzyme synthesis is controlled both directly and indirectly by multiple two-component regulatory systems. For example, *S. enterica eptA* and *arnT* gene expression is activated indirectly by the PhoQ-PhoP pair in response to limiting Mg^2+^, and directly by the PmrB-PmrA pair in response to excess Fe^3+^ [55]. *P. aeruginosa* regulation is more complex, with five two-component regulatory systems to control lipid A remodeling enzyme synthesis in response to limiting Mg^2+^ (PhoQ-PhoP and PmrB-PmrA), excess Zn^2+^ (ColS-ColR), and antimicrobial peptides (CprS-CprR, ParS-ParR, and PmrB-PmrA) [86] found.

In comparison to *S. enterica* and *P. aeruginosa*, *Xanthomonas* spp. encode a distinct subset of two-component regulatory systems implicated in lipid A remodeling. The PhoQ-PhoP two-component regulatory system is encoded by all three genera [53,86], emphasizing its central role in the response to limiting Mg^2+^ [87]. By contrast, the PmrB-PmrA and RaxH-RaxR (ColS-ColR orthologs) pairs are absent from *Xanthomonas* spp. and *S. enterica*, respectively, and the CprS-CprR and ParS-ParR pairs are absent from both [31,50]. Thus, conservation of RaxH-RaxR (ColS-ColR orthologs) plus PhoQ-PhoP implies that lipid A regulation in *Xanthomonas* spp. comprises a subset of the complex *P. aeruginosa* network.

The RaxH-RaxR pair was characterized and named in the context of *raxSTAB* operon expression [12]. Nevertheless, Rax phenotypes conferred by *raxH* and *raxR* null alleles are subtle, at least under the conditions studied, and potentially result from indirect effects. Better understanding of *Xanthomonas* two-component regulatory networks is necessary to define the relationship between the RaxH-RaxR pair and *raxX-raxSTAB* regulation.

### 4.3. Phenotypes Resulting from EptA-, LpxE-, and ArnT-Catalyzed Lipid A Remodeling

The outer membrane permeability barrier depends on strong lateral interactions between LPS molecules, mediated in large part by Mg^2+^ or Ca^2+^ ions that bridge phosphates in adjacent lipid A molecules [65]. In the mammalian pathogen *S. enterica*, limiting Mg^2+^ encountered during infection [88] induces EptA and ArnT synthesis for lipid A remodeling [55]. The resulting positively charged moieties counteract the phosphate negative charge partially (EtN) or fully (Ara4N), thereby reducing the Mg^2+^ dependence for LPS function [65]. By influencing LPS lateral interactions, different types of lipid A remodeling differentially affect the outer membrane structure, resilience, and permeability [65].

Lipid A remodeling confers two additional phenotypes, cation resistance and immune evasion [60]. First, LPS remodeled with EtN and/or Ara4N is more resistant to diverse toxic cations, including metals such as Fe^3+^ and antimicrobial peptides such as polymyxins [55]. EtN-mediated resistance to antimicrobial peptides is relatively weak compared to that conferred by Ara4N in both *S. enterica* [89] and *P. aeruginosa* [37,90], reflecting differences in molecular mass (EtN = 61 Da; Ara4N = 149 Da) and degrees of negative charge compensation [65]. Further variation comes from using PP-EtN vs P-EtN (Figure 3B vs. Figure 3D) or sugars other than Ara4N [79,80]. Plants also synthesize cationic antimicrobial peptides, and at least one of these induces ArnT synthesis in *P. aeruginosa* [91].

The second lipid A remodeling phenotype concerns interactions between LPS and host innate immune systems. In mammals, lipid A binds the TLR4/MD2 (Toll-like receptor 4/myeloid differentiation factor 2) complex that controls innate immune responses to lipid A [92,93]. Binding involves both the fatty acyl chains and the phosphates, and so remodeled lipid A forms are recognized poorly by the TLR4/MD2 complex [94]. Notably, modifications involving phosphates 1 and 4′ influence TLR4/MD2 recognition and signaling [73,81,95,96,97], and appear to help host cells differentiate between pathogenic and commensal species [98].

Plant receptors for LPS molecules are yet to be identified. The *Arabidopsis thaliana* LORE receptor-like kinase, previously suggested to detect lipid A [99], instead responds to co-purified medium-chain 3-hydroxy fatty acid metabolites [100]. Nevertheless, plant recognition of LPS is a prominent feature of the innate response to bacteria [63,99,101]. Although plant-associated bacteria remodel their lipid A upon exposure to antimicrobial peptides [91], specific roles for lipid A and its remodeling are not defined. *Xanthomonas* spp. provide relatively simple models to evaluate roles for lipid A and its remodeling in a broad range of bacterium-plant contexts.

## Figures and Tables

**Table 1:** Ortholog sequence identity and coverage in pairwise alignments.

Symbol ^1^	Query ^2^	Length	From	To	Cover	Subject	Identity	Gaps
EptA	Xoo	556	24	556	96%	Pae	45%	1%
	Pae	567	6	544	95%	Xoo	45%	1%
	Xoo	556	22	549	95%	Nmb	38%	1%
	Nmb	544	7	540	98%	Xoo	38%	2%
LpxE	Xoo	254	47	251	80%	Pae	33%	1%
	Pae	264	16	232	82%	Xoo	33%	2%
	Xoo	254	107	169	24%	Ret	29%	7%
	Ret	244	103	215	46%	Xoo	32%	11%
ArnT	Xoo	577	30	336	53%	Pae	31%	8%
	Pae	549	40	345	56%	Xoo	32%	8%
ArnC ^3^	Xoo	240	6	212	86%	Pae	30%	2%
	Pae	339	9	214	60%	Xoo	30%	2%
FlmF1 ^4^	Xoo	348	17	320	87%	Fno	38%	0%
	Fno	314	9	312	96%	Xoo	38%	0%

^1^ Locus tags and accession numbers are in Table S1. ^2^ Fno, *Francisella novicida* U112; Nmb, *Neisseria meningitidis* MC58; Ret, *Rhizobium etli* (*leguminosarum*) CFN42; Pae, *Pseudomonas aeruginosa* PAO1; Xoo, *Xanthomonas oryzae* pv. *oryzae* PXO99^A^. ^3^ Xoo gene PXO_RS22265. ^4^ Xoo gene PXO_RS14680.

## Data Availability

All data are presented in the paper.

## Supplementary Materials

The following are available online at https://www.mdpi.com/article/10.3390/microorganisms9071458/s1, Figure S1, Periplasmic domains from select RaxH and ColS sequences; Figure S2, raxRH gene neighborhoods in members of the order Lysobacterales (formerly Xanthomonadales); Figure S3, Percent G+C across the gcvP-minCDE region in Xoo PXO99A; Table S1, Locus tags and accession numbers; Table S2, Genome sequences used for gene neighborhood analysis.
