# Supplementary material for: The Xanthomonas RaxH-RaxR Two-Component Regulatory System Is Orthologous to the Zinc-Responsive Pseudomonas ColS-ColR System
Source: Microorganisms. 2021 Jul 7;9(7):1458. doi: 10.3390/microorganisms9071458 (PMC8306577; doi:10.3390/microorganisms9071458)
Supplement: Supplementary file 1 [file microorganisms-09-01458-s001.zip › microorganisms-1267916-supplementary.pdf]

Supplementary Information

The *Xanthomonas* RaxH-RaxR two-component regulatory system is orthologous to the zinc-responsive *Pseudomonas* ColS-ColR system

Valley Stewart[1] and Pamela C. Ronald[2]

[1]Department of Microbiology & Molecular Genetics
[2]Department of Plant Pathology and Genome Center
University of California, Davis

vjstewart@ucdavis.edu | pcronald@ucdavis.edu

Figure S1 (following page). Periplasmic domains from select RaxH and ColS sequences. Highlighted residues are: Glu in ExxE motifs (yellow); invariant in all sequences (black); invariant in all RaxH sequences (green); invariant in all ColS sequences (blue); approximate transmembrane segment boundaries (light gray).

Abbreviations:

Xoo	*Xanthomonas oryzae* pv. *oryzae* PXO99[A]
Sma	*Stenotrophomonas maltophilia* K279a
Aco	*Arenimonas composti* DSM 18010
Lgr	*Luteimonas granuli* Gr-4
Lyi	*Luteimonas yindakuii* S-1072
Len	*Lysobacter enzymogenes* YC36
Lgu	*Lysobacter gummosus* 3.2.11
Psp	*Pseudoxanthomonas spadix* BD-a59
Psu	*Pseudoxanthomonas suwonensis* 11-1
Rte	*Rehaibacterium terrae* DSM 25897
Tca	*Thermomonas carbonis* KCTC 42013
Cfl	*Chiayiivirga flava* DSM 2416
Dko	*Dokdonella koreensis* DS-123
Pae	*Pseudomonas aerugionosa* PAO1
Ppu	*Pseudomonas putida* KT2440
Pfl	*Pseudomonas fluorescens* A506
Psy	*Pseudomonas syringae* pv. *tomato* DC3000
Pst	*Pseudomonas stutzeri* A1501

Figure S1. Legend on previous page.

```
Xoo           MSKQRPLGLRVLVWLFGYTLLMTLAVFGTAQYLHERAEHGVWRSLLNSELDSILERSVQNPDYHWQDSDTLRLY  74
Sma          MKASKPGPLYRRVVWWLLGYLALLSIAVFSVGNYVHEHAEHAAWRALLNSELDSIVEHTEHEPHYRWQDSDTLSLF  76
Aco           MSARPASLRRRIVLWLLAFATALTVAVIAQGAFVVEYVERSVWRSLLTVELDHFLERRRTDPAFRWDGTAGIAVY  75
Lgr             MRTRRSLRRRLMQALTAYVALLSLVVLVHGYTLNERAEALVWESLLTAEFEHLLERRSEEPGYQWHDTDTLVLF  74
Lyi             MRTRPSLTRQITLGLVVYGVLLSVALFVHGLLVNEQAERMVWQAMLETSMDDLLERRRHDPDFGWRNNGRLDLH  74
Len       MATPDRPAAPARPRSSLRQRILLGLFGYTALLSIAVVVQGFFVNEHAEHLVWTSLMNTEMDYFVERSRSDPDYRWSDTQALSLY  84
Lgu  MATPAESPPDKSKPGRKTRSSLRQRILLGLFGYTALLSIAVVVQGVIVNEHAEHLVWTSLMDTELDHFVERSRIDADYRWTDTLALSLY  89
Psp           MSLPMARRISLRKRLLRWALVYLVVVTGAVVLGGEVVNEHAERQVWRSLMRSDLQHFVERRQADPGYNWIDDPAERLY  78
Psu        MKPGGIRSRLGGLRARVTLWLVIYAALISLAVFIHGFIVNEQAEQLTWRSLLKSELAHFVQRSAEDPHYHWTDTETVQLY  80
Rte              MNRRTLRARIATGLLGYALLMAAAIFLHGYIVNEAVERTVWRSLLETELDYFQTRRAADPRFEWPQTETLALY  73
Tca          MPLRQPHSLHHRIVLWLLAFALALTVAVIAQGTVVIEYVERIVWRSMLMAELDHFIQRSRNNPGYHWDETAGFVIH  76
Cfl              MIRSTLRSRIAASLFVYSLVLAAAVLSLGYLVNEDVEAVIWAAVLEPAMERHLAAEGRLETPRVSGRIGTFSV  73
Dko             MKPRWGLRRRITVGLIAYAVVLSIAVAVHGFFVNENAEHLVWESLLHAEMDHFLNRRATDPDYRWPDTQTLELL  74

Pae             MEYKQSLARRIVIAFMLMTVAVGGLFSAGIVGVVHIIEERLISRDLGGELERILRDDLAQGRNPVLDPGMRFFI  74
Ppu             MEFKQSLAQRIIIAFALMSALVAGAFAFGIVGTVHLVEERLISSVLGGDLQRLLRMDSVSDWSHRPRPDQLFYF  74
Pfl             MEFKQSLAQRIIIAFALMSALVAGAFAMGIVATVHLVEEKLISAGLGGDLQRLLLMDSVSDWHHRPEPDQLFYF  74
Psy             MEFKQSLAQRIIIAFALMSALVAGSFAIGIISTVHLVEEKLISAGLGGDLNRLMLMDSVSDWSHRPKPDQLFYF  74
Pst             MLAKQPFARRIVIAFTLMTLVVSGVFSLGIVAVVYFVESQLVTGELSRELDIVLHEDLPSGRPPRLDAQTRFFS  74

                              Transmembrane 1     ExxE


Xoo  HIDDV----ARAPPVLRTLPPGLHDGLEIAGRQSAVMVRDTAHAGRLALVLDITDFEALEKFLTRWMLAAGIALIGITLLMGTYAMARL 159
Sma  RFDE-----GNMPESVRLLHPGLHDGVPIKGRQTAVMVRDTGSMGRVALALDISDFHDLEQFATRWVMLAGVIMIFVTVLMASFGMERM 160
Aco  RGDD-----PALPPELSGLAPGLHDDLRIGAAEYVVLVRDDGGV-RHLLAMDIDGFTRDESGSEWLTVAAALLLAAIGVAAVVGVSRL 158
Lgr  GDA------RPLPPEVVALGPGLHDEVRIGDGEYVLMSREVGGD-MLALAIDIADFERSEREIAATLLGSTLLVAVLLGLFAMLGAQRL 156
Lyi  ILDAAD---ARTPDALRPLAPGLHDNVFFGDNEWVVLVREEAGI-RYALALDIDGFEDLEWELVKPVIASSVLFMLLLGIAVYFGARLL 159
Len  DSAT-----RPPPRELAALAPGVHDEVVVAGVEYVTLVRDIDGR-RLTLALDIDDMEHREFDLALTIAGSAVTTLLLLGVVIGWGVRRL 167
Lgu  DDAT-----RPPPPELRGLSPGIHDEVGYNGVEHVVLVREVDGR-RLTLALDIDDMEHREFDLALTIAGSAITTLLLLCVVIGWGVRRL 172
Psp  FLNTD----PDVPTKLAALKPGLHDDFSIDGVEHVVLVEDTPLG-RLALALDLTRFEGVEGWVTLGTVGAAAVAIAVFGLLMAWGLGRA 162
Psu  GDDDD----SPAPPEFARFGPGIHDGIEYQGRDKVLMVQDVNGR-RLVLALDITQLQAHENNLGLWMLVSNIIAIVLLGILVAWGLGRV 164
Rte  RRPLSETASAHLPLALERLPPGLHDEVALDDHEVVVLVRDIGDE-RVYLTLDISDLEREERRLLLLLVGGSLLAAGLLAVLAWVVAGRQ 161
Tca  VGADD----PDLPVELRGLKPGLHDDILVAGTNHVAMVHDDAGI-RYALVMDIEQFEIEESGFELLTLLAAVLLLLAVSVAAVFGVRRL 160
Cfl  RTAAPWP--TDVPGDLRTLEPGFHDEIRMAGREAAVLVRDGAGW-RDFVVVDITDLESAERSFAALLLGGALVAALLLVWTVHWLAGRL 159
Dko  TFPAG----STIPPPLDTLEPGVHDEFMLEGRERVVLIRDVDGQ-RLVLALDIGDLERREYDLGWALLASALAMVLVMAGAVAWGVGLL 158

Pae  SDGQGS---YAMPPALDQLDVGFHEVFEGD-LSFHALVRDIDGR-RFVLLQDQSDFEAREQVLYASVLTGYVLSIALAGLLGWMLARKV 158
Ppu  SGGRDD---FELPKDLRHLDRGFHEVFRDQ-LSYHAMVEIVDGR-RYVLLQDQSDFEERERVLFAVVVGFVLSLVLAVILGWLVARRV 158
Pfl  SGGPGD---FELPKDLRHLERGFHEVFREQ-LSYHAMVEIVDGR-HYVLLQDQSDFEERERVLFAVVLVGFVLSLALAVFLGWVLARRV 158
Psy  SNGPGD---FDLPKDLRHLEPGFHEVFRGP-LSYHAMIEVVDGR-HYALLQDQSDFEERERVLFAVVLVGFVLALALAVFLGWVLARRV 158
Pst  TALPD----YPLPEGFATLPEGFTEVVRGD-EAYYVYVRDLDGE-RYVLMEDQNEFEERENALFKTVLAGFLLSVLGAWLLGRLMAHKV 157

                                                         ExxE      Transmembrane 2
```

Figure S2 (following page). *raxRH* gene neighborhoods in members of the order *Lysobacterale*s (formerly *Xanthomonadales*). Non-contiguous genes are on separate lines. Genes are color-coded: *raxR* (dark blue); *raxH* (light blue;) *eptA*, phosphoethanolamine transferase (dark red); *lpxE*, lipid A 1-phosphatase (red); *arnT*, sugar-undecaprenyl phosphate transferase (orange); predicted undecrapenyl phosphate-sugar transferase, and amino-terminal domain in the *Chlamydia* LpxB lipid A-disaccharide synthase (green); predicted sugar nucleotide epimerase (light green); *mdoB*, phosphoglycerol transferase (dark gray); predicted methyltransferase (light gray); LPS heptose kinase (white); and insertion sequence (black rectangle).

Figure S2. Legend on previous page.

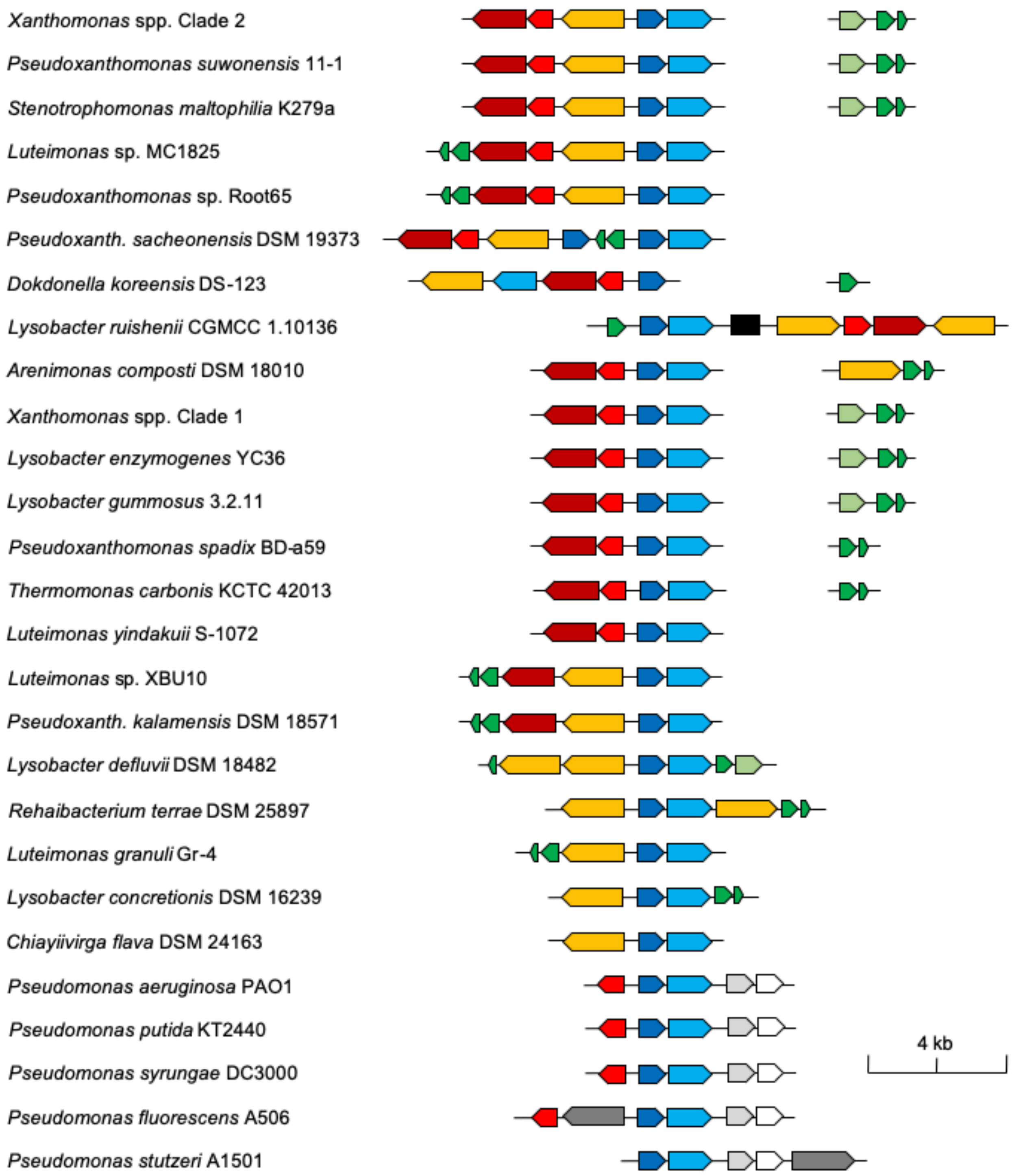

Figure S3. Percent G+C across the *gcvP-minCDE* region in *Xoo* PXO99^{A}. The plot was constructed in MegAlign. The red line shows the median G+C value for the PXO99^{A} genome.

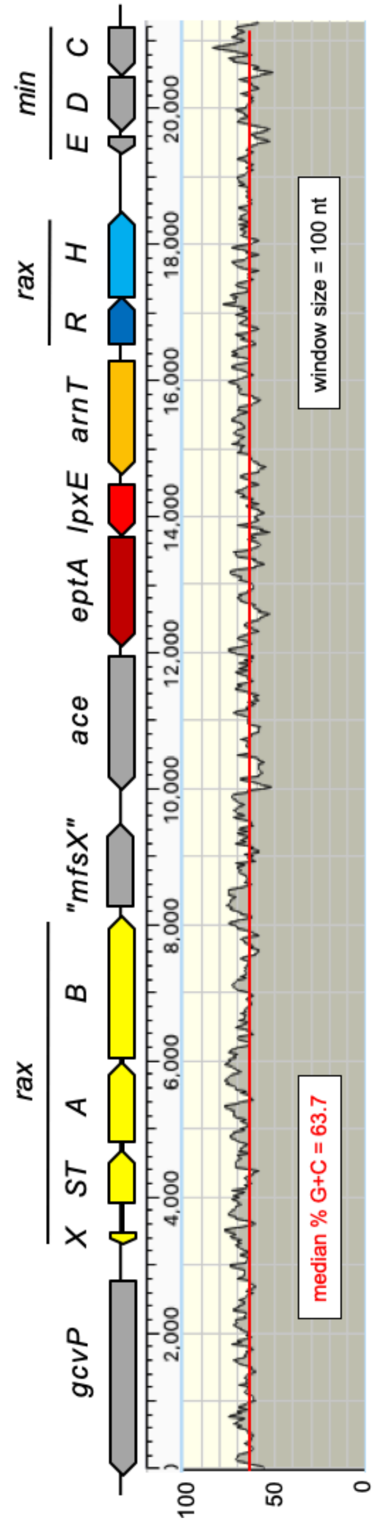

Table S1. Locus tags and accession numbers.

Symbol	Strain[a]	Locus Tag	Accession	Length
ColR	*Pae*	PA4381	WP_003094039.1	227
DetR	*Xoo*	PXORS_05125	WP_002811889.1	237
RaxR	*Xoo*	PXORS_06050	WP_011409239.1	238
VgrR	*Xoo*	PXORS_18925	WP_011407833.1	225
ColS	*Pae*	PA4380	WP_003094037.1	426
ColS	*Ppu*	PP_0902	AAN66527.2	436
ColS	*Pfl*	PSEBR_a4371	CAA70932.1	425
ColS	*Pst*	PST_0914	WP_011912104	422
ColS	*Psy*	PSPTO_4373	WP_003380966	429
DetS	*Xoo*	PXORS_05130	WP_027704202.1	423
RaxH	*Xoo*	PXORS_06055	WP_027703305.1	430
VgrS	*Xoo*	PXORS_18935	WP_027704076.1	439
EptA	*Xoo*	PXO_RS06035	WP_041182603.1	556
EptA	*Pae*	PA1972	WP_003113468.1	567
EptA	*Nmb*	NMB1638	WP_002216735.1	544
LpxE	*Xoo*	PXO_RS06040	WP_011409241.1	254
LpxE	*Pae*	PA4382	WP_003112760.1	264
LpxE	*Ret*	—	WP_011654039.1	244
ArnT	*Xoo*	PXO_RS06045	WP_027703306.1	577
ArnT	*Pae*	PA3556	WP_038403631.1	549
ArnT	*Cme*	Rmet_4828	ABF11690	575
—	Xoo	PXO_RS2226	WP_011407452.1	240
ArnC	*Pae*	PA3553	WP_003112880.1	339
—	*Xoo*	PXO_RS14680	WP_011408225.1	348
FlmF1	*Fno*	FTN_1403	ABK90273.1	314
FlmF2	*Fno*	FTN_0545	ABK89440.1	318

[a] *Cme*, *Cupriavidus metallidurans* CH34; *Fno*, *Francisella novicida* U112; *Nmb*, *Neisseria meningitidis* MC58; *Ret*, *Rhizobium etli* (*leguminosarum*) CFN42; *Pae*, *Pseudomonas aeruginosa* PAO1; *Pfl, Pseudomonas fluorescens* WCS365; *Ppu*, *Pseudomonas putida* KT240; *Psy*, *Pseudomonas syringae* pv. *tomato* DC3000; *P*st, *Pseudomonas stutzeri* A1501; *Xoo*, *Xanthomonas oryzae* pv. *oryzae* PXO99[A]

Table S2. Genome sequences used for gene neighborhood analysis.

Species	Strain	Accession
**Order *Lysobacterales* (formerly *Xanthomonadales*)**		
**Family *Rhodanobacteraceae***		
Chiayiivirga flava	DSM 24163	NZ_JACHHP000000000.1
Dokdonella koreensis	DS-123	NZ_CP015249.1
**Family *Lysobacteraceae* (formerly *Xanthomonadaceae*)**		
Arenimonas composti	DSM 18010	NZ_AUFF00000000.1
Luteimonas granuli	Gr-4	NZ_CP042218.1
Luteimonas yindakuii	S-1072	NZ_CP039383.2
Luteimonas sp.	MC1825	NZ_CP061714.1
Luteimonas sp.	XBU10	NZ_VTFT00000000.1
Lysobacter concretionis	DSM 16239	NZ_AVPS00000000.1
Lysobacter defluvii	DSM 18482	NZ_AUHT00000000.1
Lysobacter enzymogenes	YC36	NZ_CP040656.1
Lysobacter gummosus	3.2.11	NZ_CP011131.1
Lysobacter ruishenii	CGMCC 1.10136	NZ_VLKP00000000.1
Pseudoxanthomonas kalamensis	DSM 18571	NZ_PDWQ00000000.1
Pseudoxanthomonas sacheonensis	DSM 19373	NZ_PDWS00000000.1
Pseudoxanthomonas spadix	BD-a59	NC_016147.2
Pseudoxanthomonas suwonensis	11-1	NC_014924.1
Pseudoxanthomonas sp.	Root65	NZ_LMHA00000000.1
Rehaibacterium terrae	DSM 25897	NZ_JACHHX000000000.1
Stenotrophomonas maltophilia	K279a	NC_010943.1
Thermomonas carbonis	KCTC 42013	NZ_BMZL00000000.1
**Clade 1 *Xanthomonas* spp. with *raxX-raxSTAB***		
Xanthomonas translucens pv. *cerealis*	CFBP 2541	NZ_CM003052.1
Xanthomonas translucens pv. *translucens*	DSM 18974	NZ_CAPJ00000000.1
Xanthomonas translucens pv. *undulosa*	ICMP 11055	NZ_CP009750.1
**Clade 1 *Xanthomonas* spp. without *raxX-raxSTAB***		
Xanthomonas albilineans	GPE PC73	NC_013722.1
Xanthomonas hyacinthi	DSM 19077	JPLD00000000.1
Xanthomonas sacchari	R1	NZ_CP010409.1
Xanthomonas theicola	CFBP 4691	NZ_CP049017.1
**Clade 2 *Xanthomonas* spp. with *raxX-raxSTAB***		
Xanthomonas alfalfae	GEV-Rose-07	MIKD00000000.1
Xanthomonas axonopodis pv. *manihotis*	UA536	AKEQ00000000.1
Xanthomonas cucurbitae	CFBP 2542	NZ_MDED00000000.1
Xanthomonas dyei	CFBP 7245	NZ_MDEE00000000.1
Xanthomonas euvesicatoria	85-10	NC_007508.1

Table S2 (continued**).** Genomes used for gene neighborhood analysis.

Species	Strain	Accession
Xanthomonas maliensis *	M97	NZ_AQPR00000000.1
Xanthomonas perforans	91-118	NZ_CP019725.1
Xanthomonas phaseoli pv. *dieffenbachiae*	LMG 695	NZ_JPYB00000000.1
Xanthomonas phaseoli pv. *phaseoli*	CFBP 412	NZ_CP020964.2
Xanthomonas prunicola	CFBP 8353	NZ_PHKV00000000.1
Xanthomonas oryzae pv. *leersiae*	NCPPB 4346	LHUK00000000.1
Xanthomonas oryzae pv. *oryzae*	AXO1947	NZ_CP013666.1
Xanthomonas oryzae pv. *oryzae*	PXO99[A]	NC_010717.2
Xanthomonas oryzae pv. *oryzicola*	BLS256	NC_017267.2
Xanthomonas vasicola pv. *musacearum*	NCPPB 4392	AKBI00000000.1
Xanthomonas vasicola pv. *vasculorum*	NCPPB 206	AKBM00000000.1
**Clade 2 *Xanthomonas* spp. without *raxX-raxSTAB***		
Xanthomonas arboricola pv. *juglandis*	Xaj 417	NZ_CP012251.1
Xanthomonas bromi	CFBP 1976	NZ_MDCE00000000.1
Xanthomonas campestris pv. *campestris*	8004	NC_007086.1
Xanthomonas campestris pv. *raphani*	756C	NC_017271.1
Xanthomonas cannabis pv. *cannabis*	NCPPB 2877	JSZE00000000.1
Xanthomonas cassavae	CFBP 4642	NZ_ATMC00000000.1
Xanthomonas citri pv. *citri*	306	NC_003919.1
Xanthomonas codiaei	CFBP 4690	NZ_MDEC00000000.1
Xanthomonas floridensis	WHRI 8848	NZ_LXNG00000000.1
Xanthomonas fragariae	LMG 25863	AJRZ00000000.1
Xanthomonas hortorum pv. *gardneri*	ICMP 7383	NZ_CP018731.1
Xanthomonas massiliensis	SN8	NZ_FCOY00000000.1
Xanthomonas melonis	CFBP 4644	NZ_MDEH00000000.1
Xanthomonas nasturtii	WHRI 8853	NZ_LYMI00000000.1
Xanthomonas pisi	CFBP 4643	NZ_MDEI00000000.1
Xanthomonas populi	CFBP 1817	NZ_MDEJ00000000.1
Xanthomonas retroflexus	Sp953	FRDD00000000.1
Xanthomonas vesicatoria	LMG 911	NZ_CP018725.1
**Order *Pseudomonadales***		
**Family *Pseudomonadaceae***		
Pseudomonas aeruginosa	PAO1	NC_002516.2
Pseudomonas fluorescens	A506	NC_021361.1
Pseudomonas putida	KT2440	NC_002947.4
Pseudomonas stutzeri	A1501	NC_009434.1
Pseudomonas syringae pv. *tomato*	DC3000	AE016853.1

* Intermediate between clades 1 and 2
